# Dizziness Handicap Inventory in der Qualitätssicherung der Therapie vestibulärer Funktionsstörung

**DOI:** 10.1007/s00106-021-01017-0

**Published:** 2021-03-17

**Authors:** T. A. Duong Dinh, J. Wittenborn, M. Westhofen

**Affiliations:** 1 Klinik für Hals‑, Nasen‑, Ohrenheilkunde und Plastische Kopf- und Halschirurgie, Rheinisch-Westfälische Technische Hochschule Aachen, Pauwelsstraße 30, 52074 Aachen, Deutschland; 2 Klinik für Mund-, Kiefer und Gesichtschirurgie, Rheinisch-Westfälische Technische Hochschule Aachen, Aachen, Deutschland

**Keywords:** Dizziness Handicap Inventory, Vestibuläre Funktionsstörung, Schwindel, Neurootologie, Dizziness Handicap Inventory, Vestibular disfunction, Dizziness, Neurotology

## Abstract

Die Therapie peripherer vestibulärer Funktionsstörungen stellt eine Herausforderung für viele HNO-Ärzte dar. Diese besteht nicht nur in der Durchführung der differenzierten neurootologischen Diagnostik, sondern auch in der Therapie und in der Verlaufskontrolle von Patienten mit vestibulärer Funktionsstörung. Insbesondere die Qualitätssicherung solcher Therapie wird in der Regel nicht adäquat abgebildet und dokumentiert. Die deutsche, validierte Version des Dizziness Handicap Inventory (DHI) bietet eine praktikable Möglichkeit zur Evaluierung und Verlaufskontrolle der Therapieergebnisse. In einer Studie wurden die Beschwerden von Patienten, welche in der HNO-Klinik des Universitätsklinikums Aachen aufgrund einer unilateralen peripheren vestibulären Funktionsstörung behandelt wurden, prä- bzw. posttherapeutisch mittels DHI erfasst und ausgewertet. Die posttherapeutische Erhebung des DHI-Scores erfolgte telefonisch. Die Auswertung der erhobenen DHI-Scores ergab in 92 % der Fälle eine signifikante Reduzierung der Schwindelbeschwerden. Es bestätigte sich die Eignung des DHI als Instrument zur Qualitätssicherung für die Funktionsdiagnostik und die darauf folgende Therapie von vestibulären Funktionsstörungen.

## Abstract (en)

Treatment of vestibular dysfunction remains a challenge for many otolaryngologists. This is true not only for establishing the differential neurotologic diagnosis, but also for treatment and treatment monitoring in vestibular dysfunction patients. Particularly quality control of therapeutic measures is generally poorly documented. The validated German version of the Dizziness Handicap Inventory (DHI) represents a viable option for evaluation and monitoring of treatment outcomes. In this study, patients who were treated at the Otorhinolaryngology Department of the University Hospital Aachen because of unilateral peripheral vestibular disorders were asked to complete the DHI before and after treatment. Posttherapeutic DHI scores were collected by telephone. Evaluation of the DHI scores underlined the significant benefit of treatment in 92%. Furthermore, the DHI prove to be useful for documentation of patients’ disorders and treatment and thus for quality assurance.

Jacobson et al. entwickelten bereits 1990 ein 25 Fragen umfassendes Inventar zur Beurteilung von Patientenbeschwerden mit Erfassen dreier unterschiedlicher Kategorien, welches hohe Reliabilität und Reproduzierbarkeit besitzt [1]. Das Inventar weist jedoch Schwächen in der Erkennung phobischer Erkrankungen in Zusammenhang mit Schwindelbeschwerden auf. Kurre et al. entwickelten eine validierte Fassung des Inventars zur Erfassung der Lebensqualität von Patienten mit Schwindelbeschwerden [2]. Sie übersetzten das Dizziness Handicap Inventory (DHI) in die deutsche Sprache und konnten feststellen, dass die deutsche DHI-Version eine hohe Reliabilität aufweist. Eine weitere deutsche Version des DHI entwickelten Volz-Sidiropoulou et al. im Rahmen einer Studie an der HNO-Klinik der RWTH Aachen [3]. Die Studie belegt, dass das deutsche DHI ein „multidimensionales und zugleich hoch reliables Instrument zur Erfassung von alltagsrelevanten Einschränkungen durch Schwindel“ ist. In der derzeitigen aktuellen Version 3.0 beinhaltet das deutsche DHI 7 körperliche, 10 funktionelle und 8 emotionale Items. Es schließt in einem eigens entwickelten Addendum zusätzliche Fragen zur Erfassung des allgemeinen Gesundheitszustands des jeweiligen Patienten ein. Diese werden jedoch bei der Auswertung der Kategorien und Berechnung des Scores nicht berücksichtigt. In der englischen Originalversion des DHI kann man jede Antwort mit ja, manchmal oder vielleicht beantworten, wobei jeder Antwort unterschiedliche Punktwerte zugeordnet werden (ja = 4, manchmal = 2, nein = 0). Im Gegensatz hierzu bedient sich der deutsche DHI-Version 3.0 der vier verschiedenen Antwortmöglichkeiten „nie, selten, öfter oder meistens“, wobei meistens mit 4, nie mit 0 und selten bzw. öfter jeweils mit 2 Punkten gewichtet sind.

Zur Abklärung ihrer Schwindelbeschwerden stellten sich die in diese Studie eingeschlossenen Patienten von 2005–2010 in der HNO-Uniklinik der RWTH Aachen vor. Aufgrund der umfangreichen neurootologischen Funktionsuntersuchungen wurden diese zum Teil stationär durchgeführt. Dort wurde erstmalig das DHI ausgehändigt und die DHI-Scores nachgehend erfasst. Die Patienten mit gesichertem unilateralem peripher vestibulärem Funktionsdefizit wurden teilweise konservativ, teilweise konservativ und operativ, je nach Diagnose, behandelt. Die Diagnosen wurden jeweils im Rahmen der neurootologischen Diagnostik festgestellt. Die posttherapeutische Erhebung des DHI-Scores wurde telefonisch durchgeführt. Eine erneute Vorstellung der Patienten war damit nicht verbunden. Die Zielsetzung der Studie ist es, zu prüfen, ob die eingeleitete Therapie eine Auswirkung auf das Beschwerdebild der Patienten hat. In diesem Zusammenhang sollte die Möglichkeit der Fernabfrage von Beschwerden logistisch und in der Auswirkung auf ärztliche Folgeentscheidungen geprüft werden.

## Material und Methoden

In die Studie wurden Patienten (*n* = 50), welche von 2005–2010 in der HNO-Uniklinik der RWTH Aachen wegen Schwindelbeschwerden unterschiedlicher Genese ambulant und/oder stationär untersucht und behandelt wurden, eingeschlossen. Die Diagnosen der Studienpatienten umfassten BPLS, Neuropathia vestibularis, M. Menière, perilymphatische Hypertension bis hin zum funktionellen Schwindel (Tab. 1). Die therapeutischen Maßnahmen umfassten abhängig von der Diagnose Befreiungsmanöver (BPLS) bis hin zur medikamentösen und operativen Therapie. Die Patienten füllten im Rahmen der Anamnese die deutsche Version (Vers. 2.0 bzw. 3.0) des Fragebogens Dizziness Handicap Inventory (DHI) aus. Der DHI-Bogen der deutschen Version 3.0 besteht aus 25 Fragen, welche die in 3 Kategorien, funktionelle (10), körperliche Einschränkungen (7) sowie emotionale Betroffenheit (8) gegliedert sind.

**Table 1:** 

*Patienten*		
Anzahl	50 (m = 19; w = 31)	–
Alter (Jahre)	64,24 (Mittelwert)	66,5 (Median)
Weiblich	64,77 (Mittelwert)	68 (Median)
Männlich	63,37 (Mittelwert)	65 (Median)
*Altersspanne*	30–86	–
*Diagnose*		Anzahl (*n*)
M. Menière/endolymph. Hydrops	–	13
Neuropathia vestibularis	–	9
BPLS	–	6
Perilymphatische Hypertension	–	5
Okulärer Schwindel	–	5
Otolithenfunktionsstörung	–	3
Hörsturz mit vest. Bet.	–	3
Contusio labyrinthi	–	1
Schwindel nicht vestib. Genese	–	5

Damit die Aktualität der Beschwerden gewährleistet ist, beziehen sich die Interviewfragen jeweils nur auf die letzten 2 Wochen vor Befragung der Patienten. Jede Frage kann mit nie, selten, öfter oder meistens beantwortet werden. Den Antwortmöglichkeiten werden verschiedene Punktwerte zugeordnet, die abschließend aufsummiert als Score getrennt für die drei Kategorien und als Gesamtscore dargestellt werden (nie = 0, selten bzw. öfter = 2, meistens = 4). Die Erhebung der posttherapeutischen DHI-Scores erfolgte nach entsprechender Einwilligung analog telefonisch. Diese Abfrage dauerte jeweils zwischen 15 und 45 min pro Patient.

Die statistische Auswertung erfolgte mittels SPSS (Version 17, 2009, Fa. IBM Corporation, Chicago/IL, USA) und Microsoft Excel (Version 2010; Seattle, WA, USA).

Zur Signifikanzberechnung wurden der T‑Test und der Wilcoxon-Signed-Ranks-Test durchgeführt. Dies galt sowohl für den DHI-Summenscore als auch für die Subscores bezüglich der funktionellen, physischen sowie emotionalen Kategorien. Die Überprüfung auf Normalverteilung erfolgte durch den Kolmogorov-Smirnov-Test (KS-Test).

## Ergebnisse

Die Tab. 1 stellt die Daten der eingeschlossenen Patienten dar.

**Figure 1:**
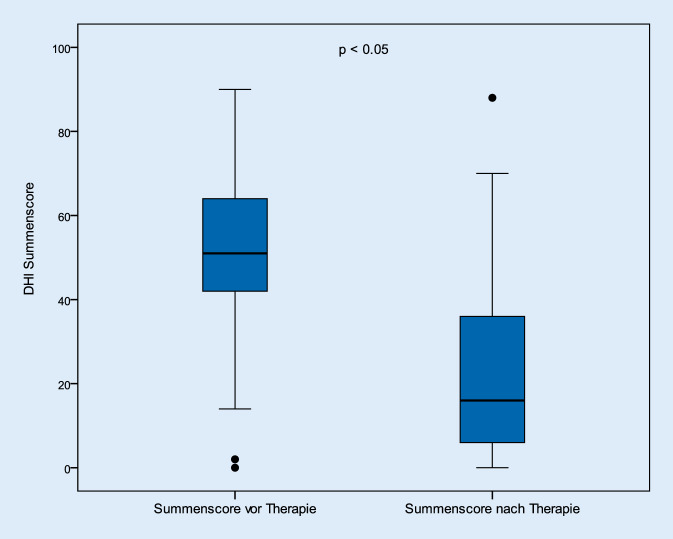

**Figure 2:**
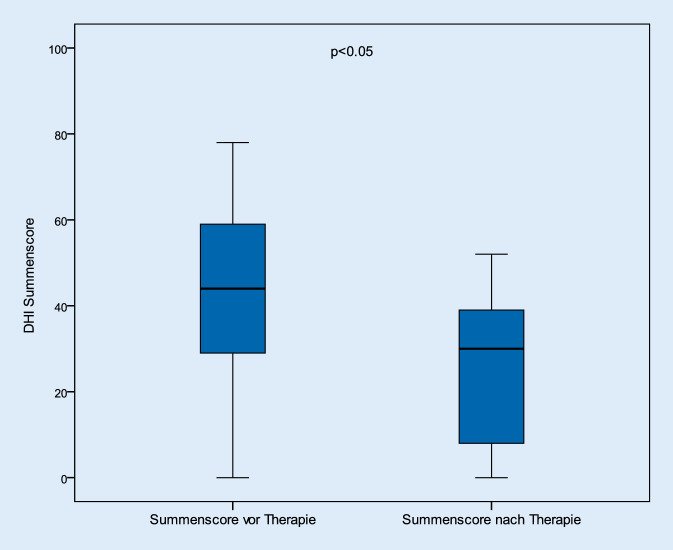

Die Abb. 1 beschreibt das Ergebnis der prä- und posttherapeutischen DHI-Summenscores. Hiernach verringerten sich die Beschwerden im Gesamtkollektiv signifikant (*p* < 0,05). Vor den jeweiligen Therapieintervallen lag dieser Wert bei durchschnittlich 50,5 (± 21,2) Punkten; nach der Behandlung betrug der Mittelwert 21,3 (± 20,3) Punkte. Betrachtet man die Patienten hinsichtlich ihres Geschlechts, so konnte kein Unterschied zwischen den beiden ermittelten Gruppen festgestellt werden. Auch hier verbesserten sich die Beschwerden nach der Therapie signifikant (Abb. 2 und 3).

**Figure 3:**
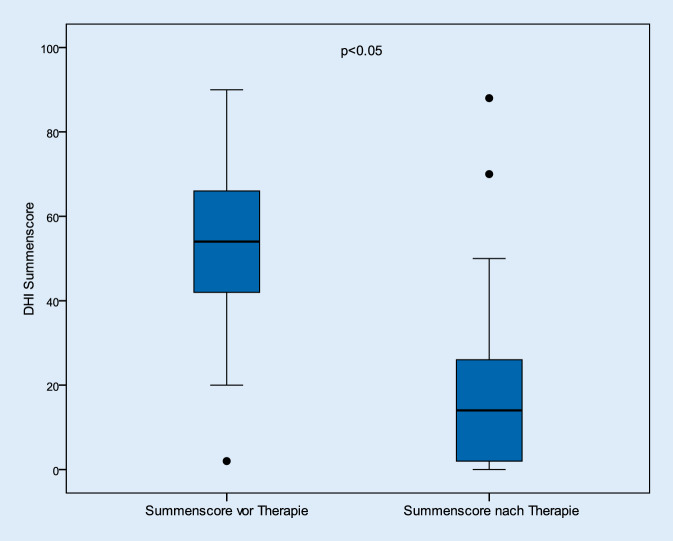

**Figure 4:**
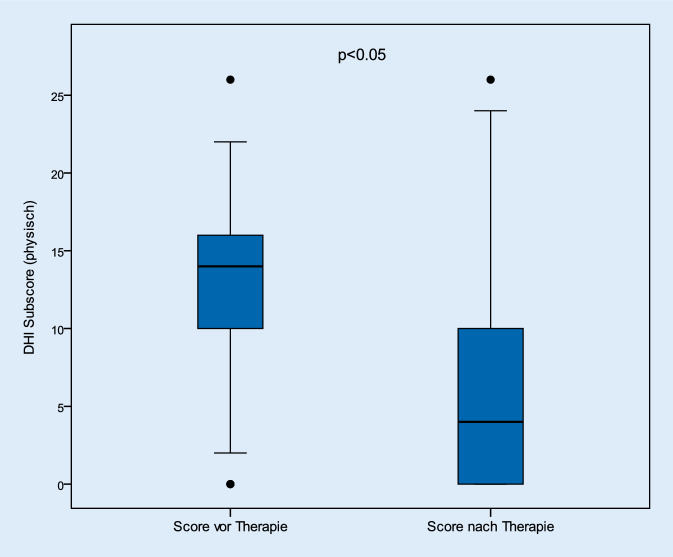

Betrachtet man die Kategorien zur Analyse der physischen, emotionalen und funktionellen Befindlichkeiten der Patienten, so konnten in gleicher Weise signifikante Verbesserungen in allen 3 Kategorien festgestellt werden (*p* < 0,05). So verbesserten sich die Beschwerden der physischen Kategorie von durchschnittlich 13,4 (± 6,4) Punkten auf 5,8 (± 6,1) nach Therapie. Auch die emotionalen und funktionellen Anteile des Gesamtscores verbesserten sich von 19,6 (± 8,3) und 17,4 (± 8,1) auf 8,4 (± 8,3) und 7,1 (± 7,4; Abb. 4, 5 und 6).

**Figure 5:**
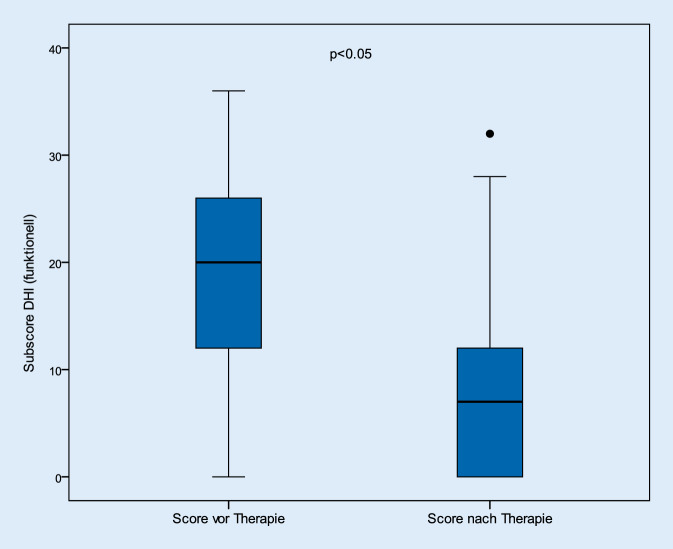

**Figure 6:**
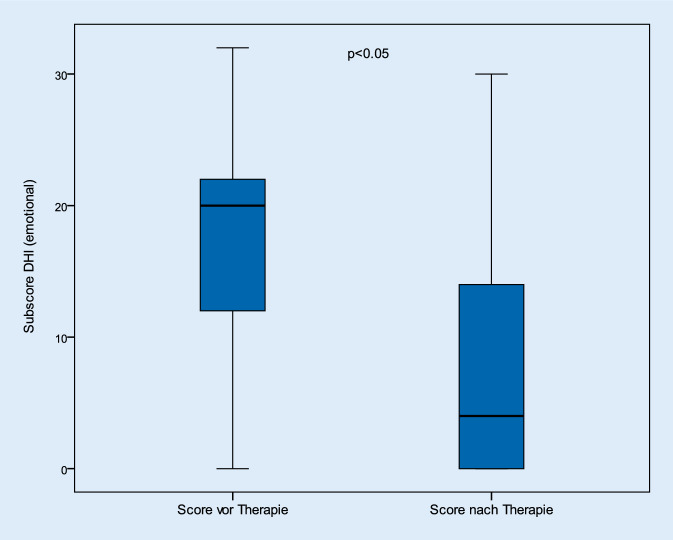

## Diskussion und Schlussfolgerung

Das Dizziness Handicap Inventory wurde mehrfach im Rahmen der Therapie vestibulärer Funktionsstörungen und der vestibulären Rehabilitation als Erfolgsindikator eingesetzt [4, 5]. So benutzen Pereira et al. 2010 die brasilianische Version des DHI zur Beurteilung des Therapieerfolgs bei Patienten mit BPLS durch das Epley-Manöver. Es zeigte sich in allen drei Subkategorien eine signifikante Verbesserung (*p* < 0,001) nach erfolgter Behandlung [4]. Auch in unserer Studie bezüglich des Therapieerfolgs bei Patienten mit BPLS konnten vergleichbare Werte bestätigt werden.

Zanardini et al. benutzten in ihrer Studie das DHI zur Beurteilung der vestibulären Rehabilitation, welche in Form von physiotherapeutischen Maßnahmen durchgeführt wurde. Auch hier konnten sie eine signifikante Verbesserung der Patientenbeschwerden darstellen [5].

Die Praktikabilität einer erneuten Befragung mittels des DHI-Inventars wird oft durch die reduzierte Compliance der Patienten, vor allem derer, die gebessert sind, eingeschränkt. Daher ist ein Bias der Ergebnisse zu befürchten. Aus diesem Grund wurde das telefonische Interview eingesetzt. Das Verschicken von Fragebögen auf postalischem Weg erscheint wegen der möglichen Rücklaufquote nicht besonders sinnvoll.

Trotz der Länge des Inventars (67 Fragen) stimmten, wie bereits oben erwähnt, 55 von 57 Patienten einer erneuten Befragung zu.

Die telefonische Erhebung des DHI stellt eine sinnvolle Alternative zur Erhebung der DHI-Scores zur Verlaufskontrolle dar. Insbesondere gilt es für Patienten, welche aufgrund der noch vorhandenen Schwindelbeschwerden oder der langen Anfahrtswegs die Klinik bzw. Praxis nicht aufsuchen kann. Diese Aufgabe kann auch von einer Krankenschwester bzw. Arzthelferin übernommen werden. Ein weiterer positiver Effekt der telefonischen Befragung schien die Tatsache zu sein, dass der Patient während der gesamten Befragung die Möglichkeit besaß, Rückfragen zu stellen und sich über den Zeitraum, auf den sich die Fragen des Inventars beziehen, zu informieren. Dadurch konnten wir zusätzlich eine vergleichbare Situation wie vor der Therapie erreichen. Der Nachteil ist u. a. eine noch komplizierte Auswertung und Berechnung des Summenscores. Zukünftig kann Software diese Aufgabe eventuell als Teil eines KI-Systems automatisch übernehmen.

Zusammenfassend stellt der Einsatz des DHI eine gute und praktikable Möglichkeit in der Qualitätssicherung der Therapie vestibulärer Funktionsstörungen dar. Zur genauen Erfassung des DHI-Scores ist auch der Zeitpunkt der Erhebung nach der Therapie notwendig. In dieser Studie spielt diese eine untergeordnete Rolle, da ein Vergleich zwischen verschiedenen Diagnosen aufgrund der geringen Patientenanzahl nicht sinnvoll erscheint. Eine vergleichbare Studie mit größerer Patientenzahl ist dafür ggf. in Zukunft notwendig.
